# Effect of Ga substitution with Al in ZSM-5 zeolite in methanethiol-to-hydrocarbon conversion†

**DOI:** 10.1039/d3ra01852k

**Published:** 2023-07-18

**Authors:** Ryo Watanabe, Natsu Oba, Suchada Smith, Kazumasa Oshima, Masahiro Kishida, Koji Miyake, Norikazu Nishiyama, Priyanka Verma, Choji Fukuhara

**Affiliations:** a Department of Applied Chemistry and Biochemical Engineering, Graduate School of Engineering, Shizuoka University 3-5-1 Johoku, Naka-ku, Hamamatsu Shizuoka 432-8561 Japan watanabe.ryo@shizuoka.ac.jp fukuhara.choji@shizuoka.ac.jp; b Department of Chemical Engineering, Faculty of Engineering, Kyushu University 744 Motooka Nishi-ku, Fukuoka-shi Fukuoka 819-0395 Japan; c Division of Chemical Engineering, Graduate School of Engineering Science, Osaka University 1-3 Machikaneyama, Toyonaka Osaka 560-8531 Japan; d Department of Chemistry, Indian Institute of Technology Delhi Hauz Khas New Delhi 110016 India

## Abstract

The catalytic properties of conventional H-[Al]-ZSM-5 and gallium-substituted H-[Ga]-ZSM-5 were evaluated in the conversion of methanethiol to ethylene (CH_3_SH → 1/2C_2_H_4_ + H_2_S). Dimethyl sulfide (DMS), aromatics, and CH_4_ were formed as byproducts on the H-[Al]-ZSM-5 catalyst. The introduction of Ga into the ZSM-5 structure provided a high ethylene yield with relatively high selectivity for olefins. Based on the temperature-programmed desorption of NH_3_ and pyridine adsorption on zeolites, the strength of acid sites was decreased by introducing Ga into the ZSM-5 structure. Undesirable reactions seemed less likely to occur at weakly acidic sites. The suppression of the formation of dimethyl sulfide (CH_3_SH → 1/2C_2_H_6_S + 1/2H_2_S) and the sequential reaction of ethylene to produce aromatics provided a high yield of ethylene over H-[Ga]-ZSM-5.

## Abstract 2

This figure is an image of the weak acid site in H-[Ga]-ZSM-5 catalyst estimated by DFT calculation.

## Graphical abstract

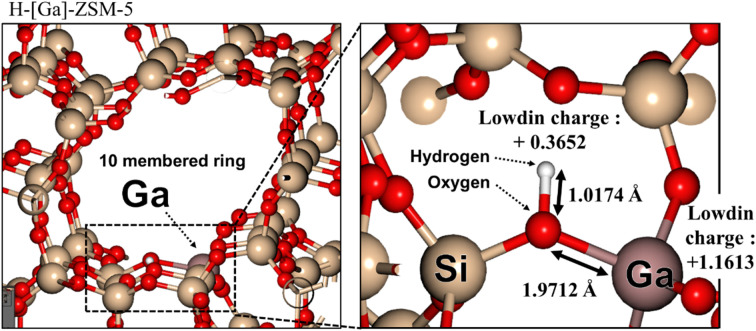

## 1. Introduction

Mercaptans (thiols) are highly reactive sulfur-containing species and have unpleasant odors. The most volatile thiol is methanethiol (CH_3_SH), a sulfur species known to be released into the atmosphere.^1,2^ A colorless gas, CH_3_SH, is found in natural gas and petroleum. In the refining process, thiols are oxidatively removed by a cobalt(ii) phthalocyanine (discovered by William Gleim and Peter Urban of UOP) catalyst.^3^ The UOP Merox™ (derived from MERcaptan OXidation) process is commercialized in over 1700 worldwide process units.^4^ Other methods of removing thiols include alkali treatment, reaction with olefins, and desulfurization, requiring oxygen, hydrogen, and olefins.^5–7^ The challenge in the removal process is to develop a novel method to convert CH_3_SH without the addition of reactants such as oxygen, hydrogen, and olefins and with minimal by-products and waste generation.

A few catalytic conversions of CH_3_SH to useful materials have been reported. Butter *et al.* used an H-ZSM-5 catalyst to convert CH_3_SH to CH_4_ and DMS at 288 °C.^8^ Mashkina *et al.* reported that acid and base catalysts with various oxides converted CH_3_SH to CH_4_ and DMS from 200 to 400 °C.^9^ Huguet recently performed a CH_3_SH conversion at low concentrations using protonated zeolites (H-ZSM-5, H–Y, and H-ferrierite) as catalysts.^10^ Above 427 °C, light alkanes (C1–C3), benzene, toluene, and xylene are formed, and carbon is deposited on the catalyst. Hulea *et al.* performed the conversions of CH_3_SH to hydrocarbon (M2TH) and CH_3_OH to hydrocarbon (MTO) to compare the reactivity of CH_3_SH with that of the similarly structured CH_3_OH.^11^ M2TH and MTO have many similarities in their selectivities toward aromatics and coke, but exhibit significant differences in selectivities toward olefins and paraffins. It has also been reported that CH_3_SH has a better methylation capacity than CH_3_OH, and that the toluene selectivity is higher for methylation to benzene using CH_3_SH.^12^ Although there are many attractive aspects of the catalytic process when CH_3_SH is used as a reaction material, most of recent studies have focused on the conversion of CH_3_SH with ppm-order concentration. For the practical application of the conversion of CH_3_SH, it is necessary to convert CH_3_SH with volume order. However, such a catalytic process has not been explored so far.

In this study, MFI-type zeolite was used as a catalyst for the conversion of CH_3_SH. To convert CH_3_SH, we focused on the catalyst for the MTO system. Recently, in MTO systems, catalysts with Ga-introduced MFI zeolite frameworks were found to be effective, where H-ZSM-5 catalysts exhibited the highest methanol conversion but low selectivity for olefins, while H-[Ga]-ZSM-5 catalysts promoted the formation of olefins, C_5_^+^, and aromatic fractions.^13^ The introduction of Ga into its framework increased the selectivity of olefins. Specifically, the Al component of the MFI-type aluminosilicate was replaced with the Ga component by hydrothermal synthesis method. We investigated the CH_3_SH conversion properties of zeolite catalysts with varying amounts of Ga component. The catalysts were characterized by physicochemical characterization *via* X-ray diffraction (XRD), N_2_ adsorption measurements, scanning electron microscopy (SEM), and evaluation of acidic properties *via* the temperature-programmed desorption (TPD) of NH_3_ and measurement of pyridine adsorption spectra using Fourier transform infrared spectroscopy (FT-IR).

## 2. Experimental

### 2.1. Zeolite preparation

The precursor solution was prepared using tetraethyl orthosilicate (TEOS), Ga(NO_3_)_3_·*n*H_2_O, Al(NO_3_)_3_·9H_2_O, 20–25 wt% tetrapropylammonium hydroxide (TPAOH) aqueous solution, and deionized water. The molar ratio was 2.0SiO_2_ : *x*T_2_O_3_ : 0.50TPAOH : 170H_2_O, where *x* varied from 0.0017 to 0.020, and T denotes Al or Ga. After stirring for 24 h at room temperature, the precursor solution was poured into a Teflon-lined autoclave and hydrothermally treated at 180 °C for 24 h. The resultant powder was mixed with deionized water and then separated *via* centrifugation. This washing process was repeated several times, and the obtained sample was dried at 80 °C. The as-made sample was calcined at 550 °C in dry air atmosphere with a heating rate of 5 °C min^−1^ for 5 h to remove the structure-directing agent. Typical ZSM-5 samples (Si/Al: 11.9, 19.5) were provided by Tosoh Corporation in Japan.

### 2.2. Characterization of zeolite

The structure of the prepared catalyst was determined *via* XRD (Ultima IV, Rigaku Co. Ltd.) using a Cu Kα radiation source. The specific surface area, pore volume, and pore size distribution were determined by N_2_ adsorption–desorption at −196 °C (3-flex: Micromeritics Instrument Co. Ltd.). Scanning electron microscopy (SEM) was performed on an electron microscope (JSM-IT700HR/LA, JEOL Co. Ltd.) operated at 15.0 kV to identify the morphology of the as-prepared zeolite catalyst.

### 2.3. Evaluation of the catalytic performance of CH_3_SH conversion

The prepared zeolites' catalytic performances, including the activity and selectivity, were examined in a conventional fixed-bed reactor. After setting 0.4 g of catalyst at the center of the quartz tube, the catalyst was dried at 500 °C in the He flow. The reaction was then performed at 400–550 °C under atmospheric pressure. The reaction condition was as follows: the CH_3_SH feed rate was 0.25 mL min^−1^ (SATP); each reaction gas flow was He/N_2_/CH_3_SH = 5.0/4.75/0.25 mL min^−1^. The gaseous reactants and products in the effluent gases (CH_3_SH, CH_3_SCH_3_, CH_4_, C_2_H_4_, C_2_H_6_, and aromatics) were collected using a micro-syringe and subsequently injected into a chromatograph equipped with thermal conductivity and flame ionization detectors (GC-8A; Shimadzu Inc., Japan) and a packed column (VZ-7, a length of 6 m, GL-Science). Conversion and yield as well as selectivity are calculated, basing on below equations.

1 CH_3_SH conv. = (*F*_CH_3_SH_ in − *F*_CH_3_SH_ out)/*F*_CH_3_SH_ in ×100

2 Yield = (*F*_product_ out × *n*_i_)/*F*_CH_3_SH_ in ×100

3 Sel. =(*F*_product_ out × *n*_i_)/(*F*_CH_3_SH_ in − *F*_CH_3_SH_ out) ×100

Here, *F*_product_ out represents the flow rate of the product in the effluent gas, *n*_i_ represents the carbon number of the product, *F*_CH_3_SH_ represents the amount feed rate of CH_3_SH.

### 2.4. Characterization of acid property

Acidic sites of the zeolites was characterized by temperature-programmed desorption of ammonia (NH_3_-TPD) using a BELCAT-A (Microtrac BEL) instrument. After the activation treatment at 500 °C in N_2_ flow for 2 h, ammonia gas (20% NH_3_/Ar at 30 mL min^−1^) was supplied for 60 min at 50 °C. After purging with He, the temperature was increased to 500 °C at a rate of 10 °C min^−1^ in He flow, and the desorption of NH_3_ was detected by a thermal conductive detector (TCD). To investigate of acidic sites in zeolite samples (H-[Al]-ZSM-5, H-[Ga]-ZSM-5) in more detail (distinguish between Brønsted and Lewis acid sites), the pyridine-adsorbed IR spectrum was performed with an FT-IR spectrometer (Frontier, PerkinElmer). Prior to the measurement, the zeolite sample was heated at 500 °C for 30 min under a vacuum of 10 Pa. And then, the adsorption of pyridine diluted by N_2_ (pyridine pressure: 2.65 kPa) was performed at 50 °C for 20 min. The adsorbed pyridine was removed at 50 °C for 30 min at 10 Pa.

### 2.5. DFT calculation

To estimate the nature of acidic sites in H-[Ga]-ZSM-5 zeolite, *ab initio* calculations were performed using the first-principles calculation code “Quantum ESPRESSO.” Projector-augmented wave (PAW) pseudopotentials were used to describe the core electrons. A plane-wave cutoff energy of 350 eV was selected, and a 289-atom unit cell for zeolite and 1 × 1 × 1 *k*-point mesh was used. The positions of the atoms and lattice parameters of each cell were optimized.

## 3. Results and discussion

### 3.1. Evaluation of physicochemical properties of Ga-incorporated MFI zeolite

**Fig. 1:**
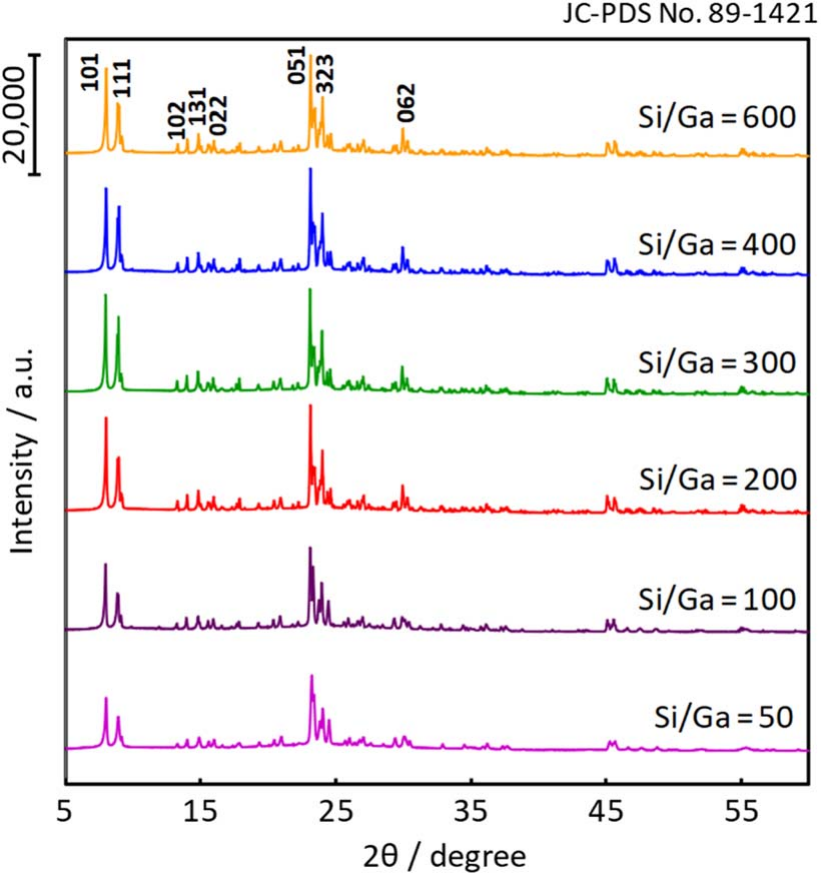
XRD patterns of H-[Ga]-ZSM-5 with various Si/Ga ratios.

Fig. 1 shows the XRD patterns of the various Ga-incorporated zeolite (denoted as H-[Ga]-ZSM-5) catalysts synthesized by the hydrothermal method. The Ga-doped samples have characteristic peaks at 5–10° and 20–25° in the 2*θ* range, which was attributed to the MFI-type zeolite. The result suggested that the crystalline structure of the ZSM-5 zeolite was preserved even after the addition of Ga species in the MFI structure.

**Fig. 2:**
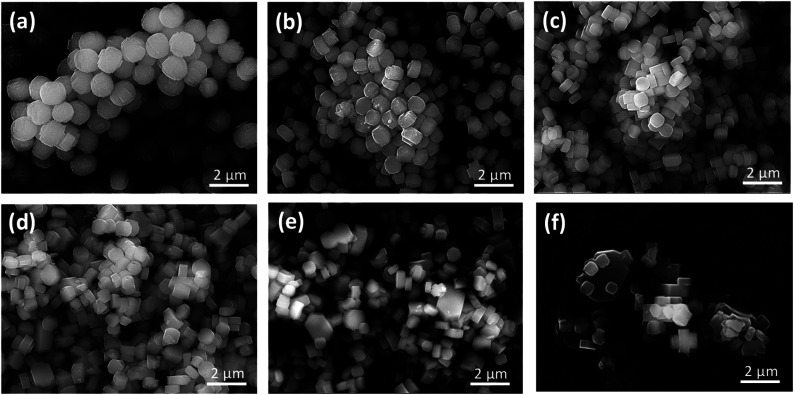
SEM images of the H-[Ga]-ZSM-5 with the Si/Ga ratio: (a) 50, (b) 100, (c) 200, (d) 300, (e) 400, (f) 600.

Fig. 2 shows SEM images of the as-prepared H-[Ga]-ZSM-5 with Si/Ga ratios of 50, 100, 200, 300, 400, and 600. Particles of H-[Ga]-ZSM-5 with Si/Ga of 50 and Si/Ga of 100 showed spherical crystals consisting of plate-like units with a particle size of about 1.5 μm. Such a spherical shape has been reported in Ga-substituted ZSM-5.^14,15^ This morphology could be attributed to the deposition of small crystals from secondary nucleation on top of the initially formed larger units, retaining the contours of the original morphology.^16^ When Si/Ga exceeded 200, the particle size gradually decreased to around 0.8 μm and the shape became a typical-hexagonal crystals of MFI-type zeolite.

**Fig. 3:**
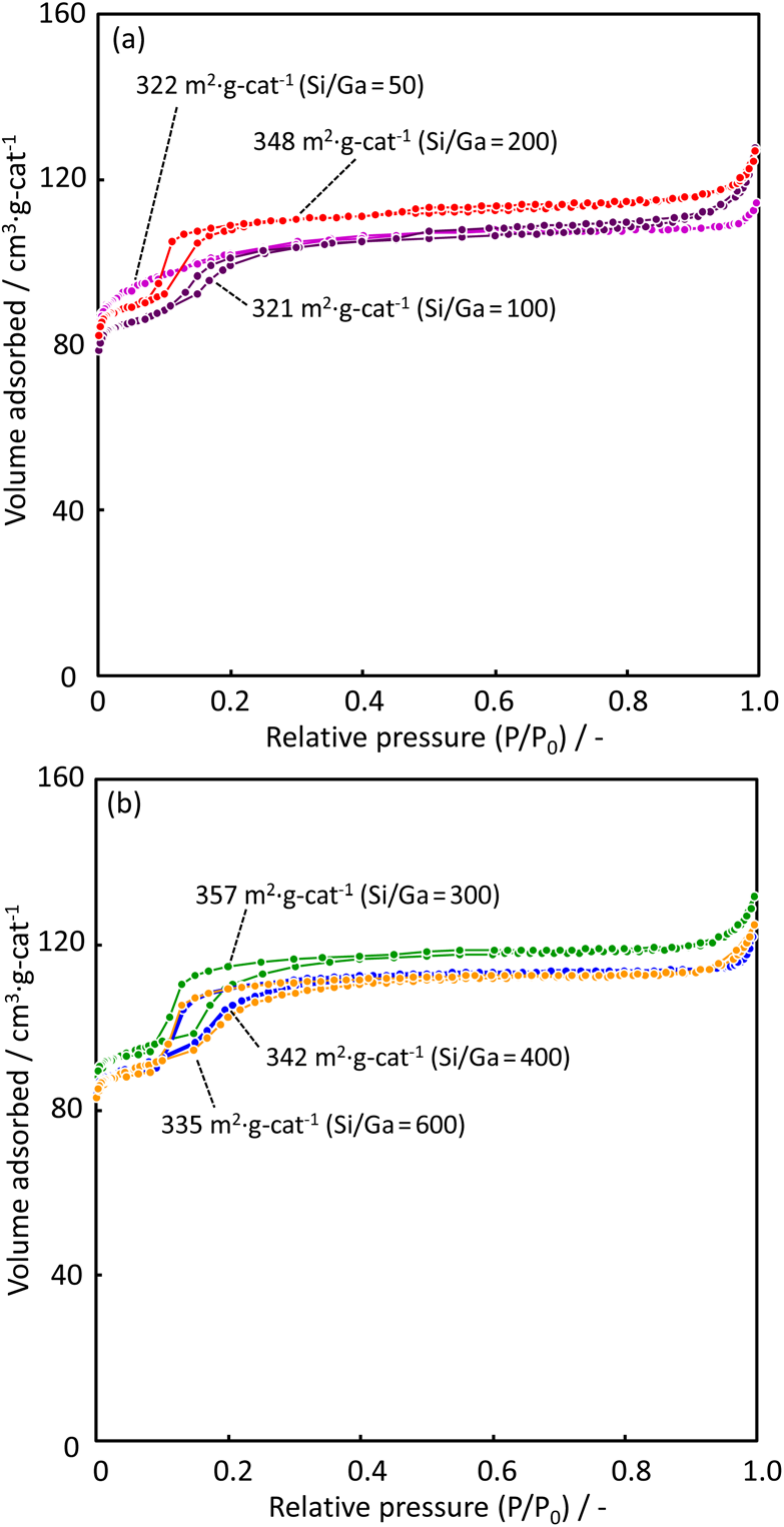
N_2_ adsorption–desorption isotherms of H-[Ga]-ZSM-5 with (a) Si/Ga = 50, 100, 200 and (b) Si/Ga = 300, 400, 600.

Fig. 3 shows N_2_ adsorption–desorption isotherms for zeolite catalysts with various Ga contents. The BET surface area of each catalyst is shown in Fig. 3(a) and (b). The adsorption isotherms are slightly different at a relative pressure of approximately 0.15. The H-[Ga]-ZSM-5 with Si/Ga > 100 catalyst has the characteristics of type I and IV isotherms. A steep uptake corresponds to micropore filling in the low-pressure region, followed by a hysteresis loop with increasing N_2_ pressure. This indicates the presence of both micropores and mesopores in the H-[Ga]-ZSM-5 zeolite. The lesser the amount of Ga introduced, *i.e.*, the higher the Si/Ga ratio, the more step-like N_2_ adsorption is observed in the catalyst. ESI† shows the pore-size distribution of H-[Ga]-ZSM-5 catalysts. The catalyst with a higher Si/Ga ratio indicates narrow pore size distribution in the micro-pore region. An increase in the N_2_ content above a relatively high pressure of approximately 0.91 was observed in all samples, indicating the possibility of the presence of macropores in H-[Ga]-ZSM-5 catalysts.^17^

**Fig. 4:**
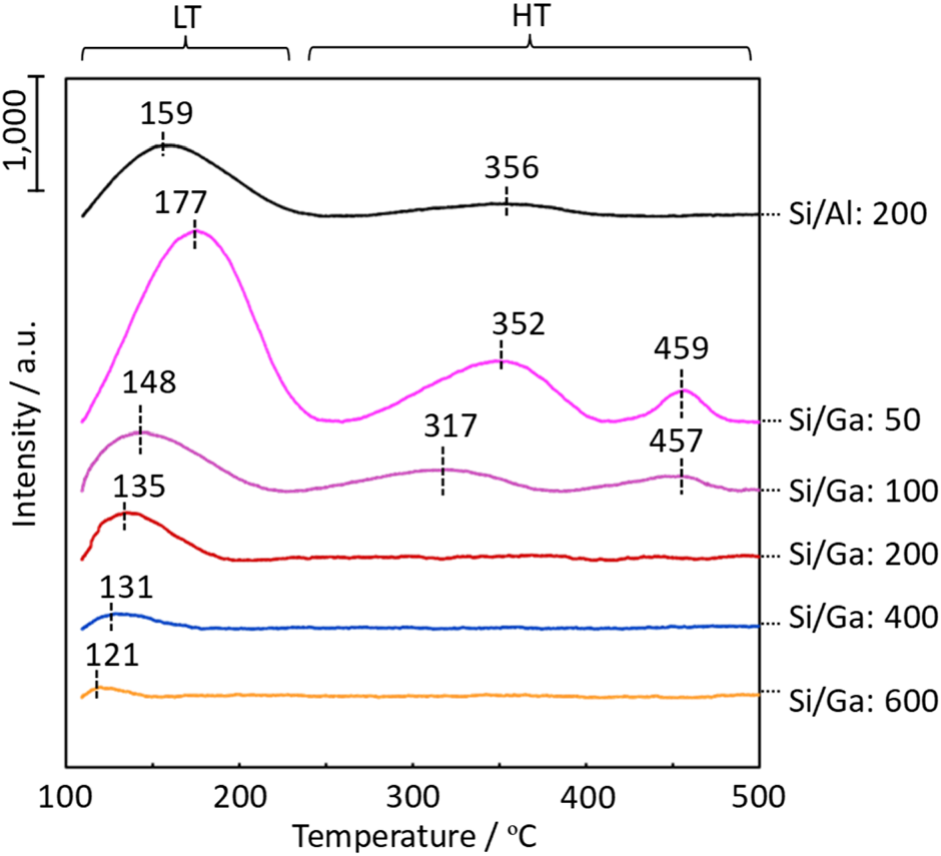
TPD profiles of H-[Al]-ZSM-5 with Si/Al = 200, and H-[Ga]-ZSM-5 with Si/Ga = 50, 100, 200, 400, and 600.

The acidic property of the H-[Ga]-ZSM-5 zeolite was evaluated to infer the change in the acidity and location of Ga in the zeolite structure. Specifically, NH_3_ was adsorbed on the zeolite catalyst at 50 °C, following which the properties, such as strength and amount of acid sites, were measured from its desorption behavior. Fig. 4 shows the TPD profiles of H-[Al]-ZSM-5 with Si/Al = 200 and H-[Ga]-ZSM-5 with Si/Ga = 50, 100, 200, 400, and 600. The peak maximum temperature clearly represents the desorption of NH_3_. Compared to Al-ZSM-5 (Si/Al = 200), the NH_3_ desorption temperature gradually decreases by decreasing the amount of Ga introduced. In addition, on the zeolite surface, the desorption of NH_3_ adsorbed by weak interactions (abbreviated as LT), and the desorption of NH_3_ adsorbed relatively strongly at high temperatures (abbreviated as HT), are observed. The peak areas of the LT and HT phenomena for H-[Ga]-ZSM-5 (Si/Ga ≤ 200) catalysts were smaller than those of Al-ZSM-5 (Si/Al = 200). The intensity of LT is considered to be proportional to the intensity of the HT in TPD spectra.^18^ In the case of H-ZSM-5, the LT peak is thought to be caused by weak acidic silanol groups or by extra-framework aluminum oxide species.^19–24^ Considering H-[Ga]-ZSM-5 with high Ga incorporation (= low Si/Ga), it has been stated that Ga can either exist as GaO, in an aggregated form on the external zeolite surface, as small particles within the zeolite pore, as an oxidizing agent GaO^+^, as a reducing Ga^+^ species, or as GaH_2_. For example, the ion exchange, impregnation, physical admixture, and chemical vapor deposition of GaCl_3_ often produce several Ga species.^25–29^ In ZSM-5 with higher Ga incorporation (= low Si/Ga), all Ga was not incorporated into the structure, and extra-framework Ga species and structural defects were produced, which might enable NH_3_ desorption on the LT side. In contrast, in Ga-ZSM-5 (Si/Ga = 200), where the LT and HT areas are small, it is expected that most of the Ga is incorporated into the framework due to the small amount of Ga, resulting in a smaller LT area. In addition, the shift to LTs at high Si/Ga ratios is assumed to be due to the weakening of the acidity. Furthermore, based on the TPD profile, the number of acid sites in Ga-ZSM-5 (Si/Ga = 400, 600) was decreased.

**Fig. 5:**
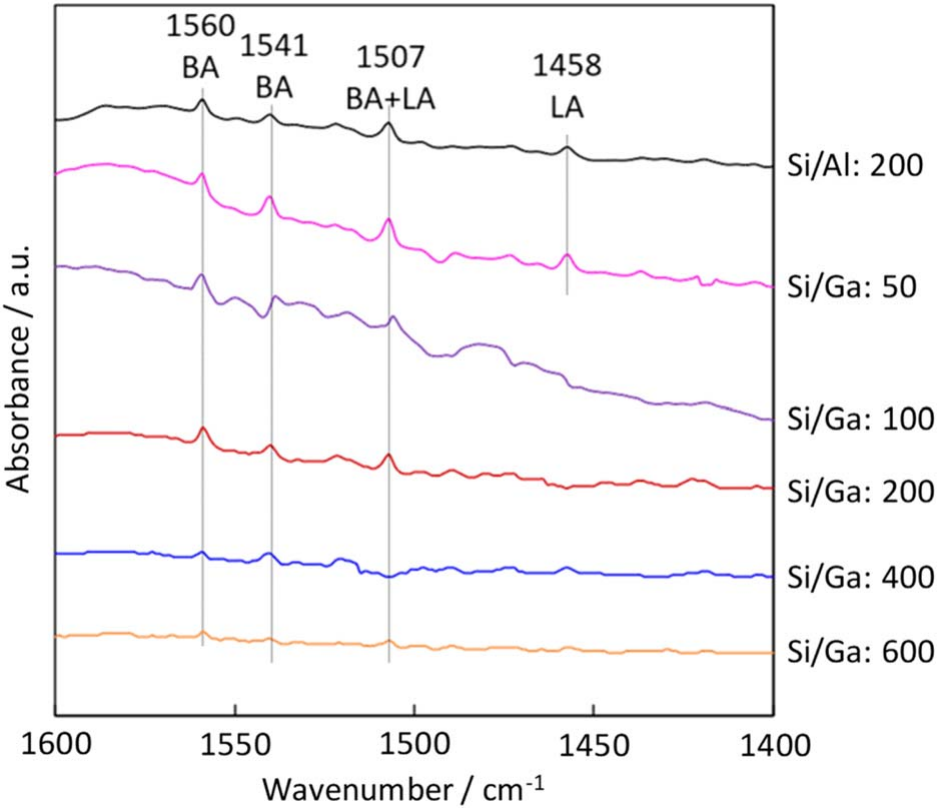
Pyridine adsorbed FT-IR spectra of the H-[Al]-ZSM-5 (Si/Al: 200) and H-[Ga]-ZSM-5 (Si/Ga: 50, 100, 200, 400, 600).

To further investigate the acidic properties of H-[Ga]-ZSM-5, FT-IR measurement was performed. Pyridine is used as a probe molecule for determining Brønsted acid (BA) sites and Lewis acid (LA) sites. The band at 1440–1470 cm^−1^ was reported to be assigned to adsorbed pyridine on LA sites, and the 1520–1560 cm^−1^ band was assigned to the protonated pyridinium ion on BA sites.^30,31^ FT-IR peak at 1458 cm^−1^ was attributed to pyridine interacting with the LA site in H-[Al]-ZSM-5 and H-[Ga]-ZSM-5 with Si/Ga of 50. While for catalysts with Si/Ga greater than 100, only the BA sites were identified. The disappearance of the LA sites with higher Si/Ga ratios might indicate the successful incorporation of Ga into the MFI zeolite framework (Fig. 5).

### 3.2. Methanethiol conversion characteristics

**Fig. 6:**
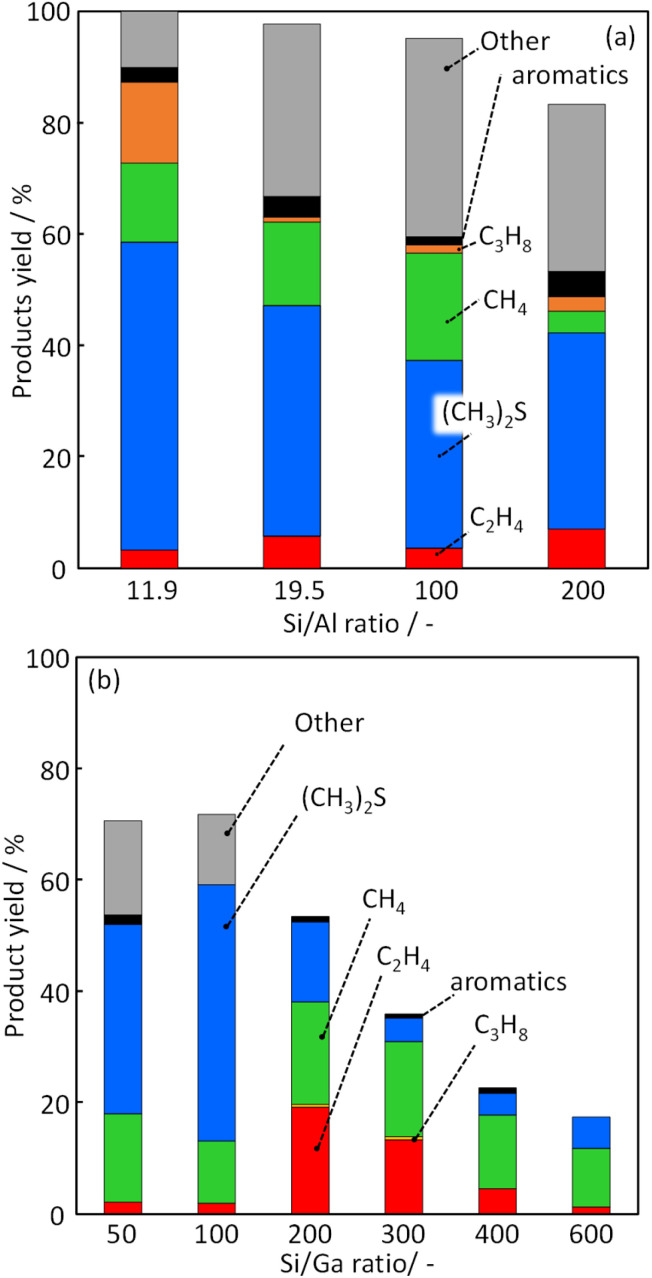
Product yield in CH_3_SH conversion over (a) H-[Al]-ZSM-5 with various Si/Al ratios and (b) H-[Ga]-ZSM-5 with various Si/Ga ratios.

To investigate the effect of Ga incorporation in the MFI zeolite framework for CH_3_SH conversion, catalytic activity tests were performed using H-[Ga]-ZSM-5 with different Si/Ga ratios. The C mol% yields of each product at a reaction temperature of 500 °C are shown in Fig. 6. The high product yield was obtained for all catalysts, but the total yield was decreased as Si/Ga ratio was increased: 70.6% (Si/Ga: 50); 71.8% (Si/Ga: 100); 53.0% (Si/Ga: 200); 36.1% (Si/Ga: 300); 22.7% (Si/Ga: 400); 18.2% (Si/Ga: 600). This decrease can be attributed to the decrease in the number of acid sites. The main products were DMS, methane, and other compounds, including unquantified trimethylbenzene, naphthalene, *etc.* (denoted as “Other”). DMS is a product obtained by the following reaction (eqn (4)), which is unsuitable for olefin production.

4 CH_3_SH → 1/2CH_3_SCH_3_ + 1/2H_2_S

CH_4_ is assumed to have been generated during the decomposition of DMS. According to the report by Ohshima, DMS decomposition occurred catalytically on the acid site.^32^ The proposed mechanism was as follows:

5 (CH_3_)_2_S + H^+^-a → CH_3_SH + (CH_3_)^+^-a

6 CH_3_SH + (CH_3_)^+^-a → H_2_S + CH_4_ + C + H^+^-a

The reaction (2) shows DMS decomposition to CH_3_SH over the Brønsted acid site, and reaction (3) shows CH_3_SH decomposition to H_2_S and CH_4_. These reactions proceed sequentially over the Brønsted acid site, and CH_3_SH formation shows the behavior of the primary product. For the H-[Ga]-ZSM-5 catalyst, the yields for other products (*i.e.*, “other yields”) were high for Si/Ga = 50 and Si/Ga = 100. However, the catalysts with Si/Ga ratio higher than 200 showed a significant decrease in the other product's yield, an increase in the ethylene yield, and a decrease in the DMS yield. According to Baltrusaitis, it was estimated by DFT calculations that the main product, ethylene, is formed *via* the formation of trimethylsulfonium ion as the key reaction intermediate.^33^ A relatively high ethylene yield was considered to be obtained by the formation of trimethylsulfonium ion, suppression of the reaction (eqn (4)) and sequential reaction to other compounds containing polycyclic aromatic molecules.

**Fig. 7:**
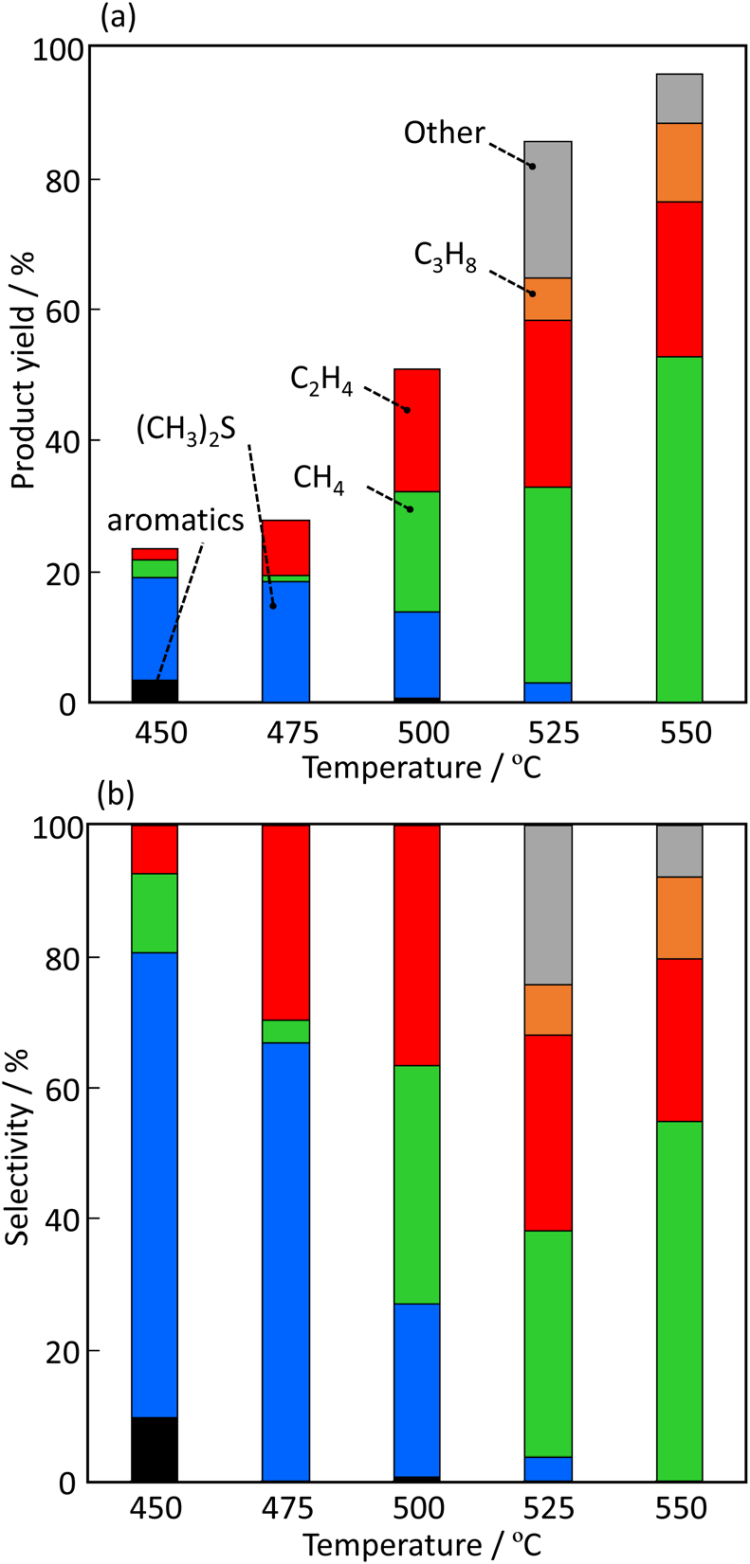
Effect of temperature on the (a) product yield and (b) selectivity over H-[Ga]-ZSM-5 with Si/Ga = 200.

Fig. 7 shows the effect of temperature on the product yield (C mol%) and selectivity over H-[Ga]-ZSM-5 with Si/Ga = 200. The yields of CH_4_ and C_2_H_4_ increased with increasing temperature. No other products were observed over Ga-ZSM-5 from 400 to 500 °C. However, the formation of other substances was confirmed at temperatures ≥525 °C. This is probably because of the polymerization of the produced ethylene to form carbon precursor species. The formation of C3 compounds was also observed at higher temperatures, which is also assumed to be due to the ETP reaction progressing over the zeolite acidic site.

### 3.3. Creation of a weak acid site on H-[Ga]-ZSM-5

**Fig. 8:**
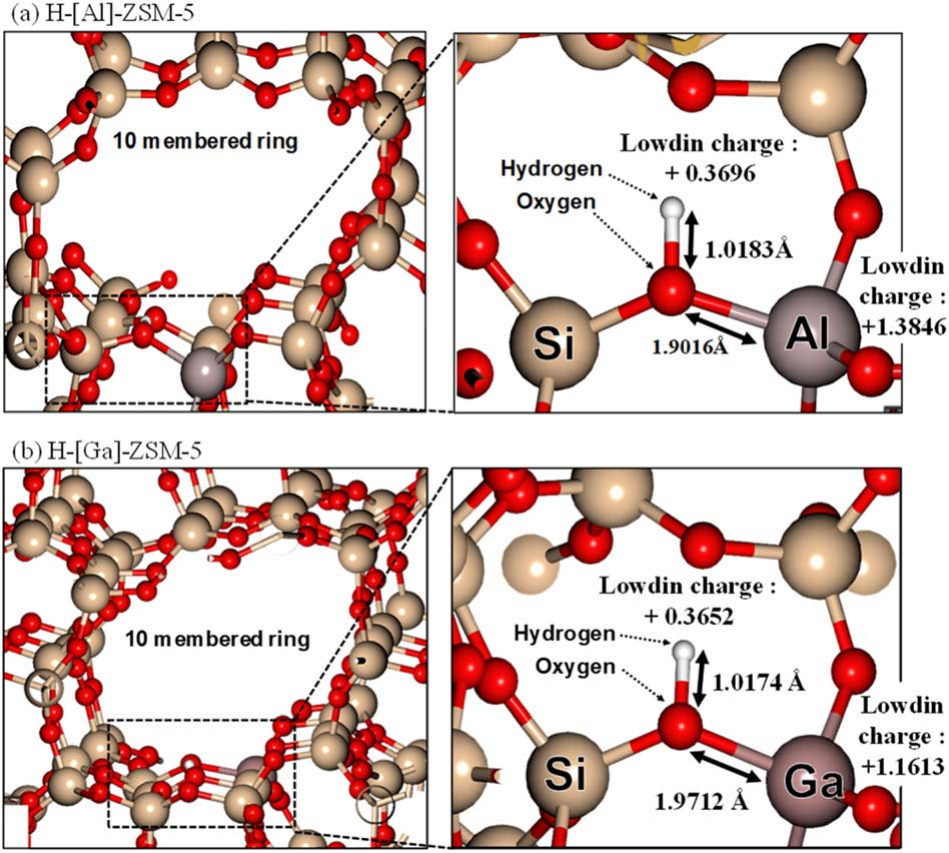
DFT calculations of (a) H-[Al]-ZSM-5 and (b) H-[Ga]-ZSM-5. (Left) Overview of 10-membered ring structure of MFI. (Right) Extended part of Al and Ga substitution in the Si site.

To estimate the property of acidic sites of H-[Ga]-ZSM-5 by calculating the optimum structure, *ab initio* calculations were performed using the first-principles calculation code “Quantum ESPRESSO.” Fig. 8 shows the local schematic of the optimized structures of H-[Al]-ZSM-5 and H-[Ga]-ZSM-5, where the Al (or Ga) atom is substituted with a Si atom in the 10-membered ring of ZSM-5. The bond distances and Lowdin charges calculated by local charge density analysis are also shown. The O–H distances of the optimized structures are 1.0174 Å for H-[Al]-ZSM-5 and 1.0183 Å for H-[Ga]-ZSM-5. In addition, the Al (or Ga)–O distance is 1.9016 Å for H-[Al]-ZSM-5 and 1.9712 Å for H-[Ga]-ZSM-5. Katada *et al.* investigated the correlation between the Al–O distance in zeolite and ammonia adsorption energy to calculate the ammonia adsorption energy (*E*_ads_) by the DFT.^34^ They proposed that a shorter Al–O distance provides an increase in the strength of acid sites of the Si(OH)Al (or Ga) unit, which would show higher ammonia adsorption energy. Based on our results, the Ga–O distance was larger than the Al–O distance, indicating that the strength of acid sites was lowered by Ga substitution with Al in the MFI framework. The charge states of the H atom in the optimized structure were +0.3696 for H-[Al]-ZSM-5 and +0.3652 for H-[Ga]-ZSM-5. In addition, the charge states of Al (or Ga) in the optimized structure were +1.3846 for H-[Al]-ZSM-5 and +1.1613 for H-[Ga]-ZSM-5. Based on these approximate values, the incorporation of Ga into the framework could decrease the strength of acidic sites, which was consistent with previously reported phenomena in the literature.

To consider the role of Ga in ZSM-5, we focused on the by-product of DMS. It has been proposed that the production of DMS is related to the Brønsted acid property according to eqn (7) and (8). The reaction proceeds *via* a carbonium ion mechanism.^9^

7 CH_3_SH + H^+^ → CH_3_SH_2_^+^

8 CH_3_SH_2_^+^ + CH_3_SH → H^+^ + (CH_3_)_2_S + H_2_S

The protonation reaction (eqn (7)) is suppressed due to the low acid strength by substituting Al for Ga, which decreases the formation of DMS. Furthermore, the decrease in the number of strong acid sites due to coking during the decomposition of DMS to CH_4_ on the zeolite might have reduced the amount of CH_4_ production.^35–37^ Additionally, the weakening of the property of the acidic sites by substituting Al for Ga suppresses the sequential reaction of ethylene to produce coke on the catalyst. The relatively good performance of H-[Ga]-ZSM-5 (Si/Ga = 200) is assumed to be due to its ability to suppress the formation of by-products and the sequential reaction of ethylene, thereby producing ethylene even with low acidity.

## 4. Conclusions

With the aim of industrial application of CH_3_SH, we investigated the reaction characteristics of gallium-substituted zeolite (H-[Ga]-ZSM-5) catalysts under conditions of high raw material (CH_3_SH) concentrations. DMS, aromatics, and CH_4_ were formed as byproducts on the H-[Al]-ZSM-5 as the reference catalyst. The introduction of Ga into the ZSM-5 structure provided a high ethylene yield of 53.0% with relatively high selectivity of 36.2%. Based on the TPD of NH_3_ and DFT calculations, the strength of acid sites was decreased by introducing Ga into the ZSM-5 structure. Undesirable reactions such as the formation of DMS and the sequential reaction of ethylene to produce aromatics seemed less likely to occur at weakly acidic sites, which provided a high ethylene yield on H-[Ga]-ZSM-5.

## Conflicts of interest

There are no conflicts to declare.

## Supplementary Material

RA-013-D3RA01852K-s001

## References

[cit1] Andersson F. A. T., Karlsson A., Svensson B. H., Ejlertsson J. (2004). J. Air Waste Manage. Assoc..

[cit2] Carlsson A. F., Rajani J. B. (2005). Hydrocarbon Eng..

[cit3] Gleim W. and Urban P., *US Pat.*, 2882224, 1959

[cit4] Brown K. M., Gleim W. K. T. (1959). Oil Gas J..

[cit5] Briot P. , Cadours R., Drozdz S. and Lecomte F., *US Pat.*, 0193925, 2007

[cit6] Carlsson A. and van Heeringen G. J., *US Pat.*, 00447201, 2009

[cit7] Mom R. V., Louwen J. N., Frenken J. W., Groot I. M. (2019). Nat. Commun..

[cit8] Butter S. A. , Jurewicz A. T. and Kaeding W. W., *US Pat.*, 3894107, 1975

[cit9] Mashkina A. V., Grunvald V. R., Nasteka V. I., Borodin B. P., Yakovleva V. N., Khairulina L. N. (1990). React. Kinet. Catal. Lett..

[cit10] Huguet E., Coq B., Durand R., Leroi C., Cadours R., Hulea V. (2013). Appl. Catal., B.

[cit11] Hulea V., Huguet E., Cammarano C., Lacarriere A., Durand R., Leroi C., Cadours R., Coq B. (2014). Appl. Catal., B.

[cit12] Cammarano C., Gay E., Finiels A., Fajula F., Hulea V. (2018). ACS Catal..

[cit13] Han Z., Zhou F., Liu Y., Qiao K., Ma H., Yu L., Wu G. (2019). J. Taiwan Inst. Chem. Eng..

[cit14] Su X., Fang Y., Bai X., Wu W. (2019). Ind. Eng. Chem. Res..

[cit15] Jin Y., Zong L., Wang X., Wei H. (2022). ACS Omega.

[cit16] Gabelica Z., Blom N., Derouane E. G. (1983). Appl. Catal..

[cit17] Sing K. (2001). Colloids Surf., A.

[cit18] Hidalgo C. V., Itoh H., Hattori T., Niwa M., Murakami Y. (1984). J. Catal..

[cit19] Lok B. M., Marcus B. K., Angell C. L. (1986). Zeolites.

[cit20] Topsøe N. Y., Pedersen K., Derouane E. G. (1981). J. Catal..

[cit21] Meshram N. R., Hegde S. G., Kulkarni S. B. (1986). Zeolites.

[cit22] Woolery G. L., Kuehl G. H., Timken H. C., Chester A. W., Vartuli J. C. (1997). Zeolites.

[cit23] Catalysis and Adsorption by Zeolites, in Studies in Surface Science and Catalysis, Elsevier, ed. H. G. Karge, G. Öhlmann, H. Pfeifer and R. Fricke, Amsterdam, 1991, vol. 65, p. 133

[cit24] Karge H. G., Dondur V. (1990). J. Phys. Chem..

[cit25] El-Malki E. M., van Santen R.
A., Sachtler W. M. H. (1999). J. Phys. Chem. B.

[cit26] Dooley K. M., Chang C., Price G. L. (1992). Appl. Catal., A.

[cit27] Price G. L., Kanazirev V. (1990). J. Catal..

[cit28] Kwak B. S., Sachtler W. M. H. (1994). J. Catal..

[cit29] Choudhary V. R., Mantri K., Sivadinarayana C. (2000). Microporous Mesoporous Mater..

[cit30] Chen Y. Y., Chang C. J., Lee H. V., Juan J. C., Lin Y. C. (2019). Ind. Eng. Chem. Res..

[cit31] Hemmann F., Agirrezabal-Telleria I., Jaeger C., Kemnitz E. (2015). RSC Adv..

[cit32] Oshima K., Kadonaga R., Shiba M., Sohmiya M., Satokawa S. (2020). Int. J. Hydrogen Energy.

[cit33] Baltrusaitis J., Bučko T., Michaels W., Makkee M., Mul G. (2016). Appl. Catal., B.

[cit34] Katada N., Suzuki K., Noda T., Sastre G., Niwa M. (2009). J. Phys. Chem. C.

[cit35] Oshima K., Kadonaga R., Shiba M., Sohmiya M., Satokawa S. (2020). Int. J. Hydrogen Energy.

[cit36] Chen S., Wang W., Zhang Y., Wei Y., Fang W., Yang Y. (2012). J. Mol. Catal. A: Chem..

[cit37] Shimoda N., Koide N., Kasahara M., Mukoyama T., Satokawa S. (2018). Fuel.

