# Supplementary figures and images for: Effect of Ga substitution with Al in ZSM-5 zeolite in methanethiol-to-hydrocarbon conversion
Source: RSC Adv. 2023 Jul 18;13(31):21441–7. doi: 10.1039/d3ra01852k (PMC10352641; doi:10.1039/d3ra01852k)

Supporting data 1 Pore size distribution in H-[Ga]-ZSM-5 catalysts.

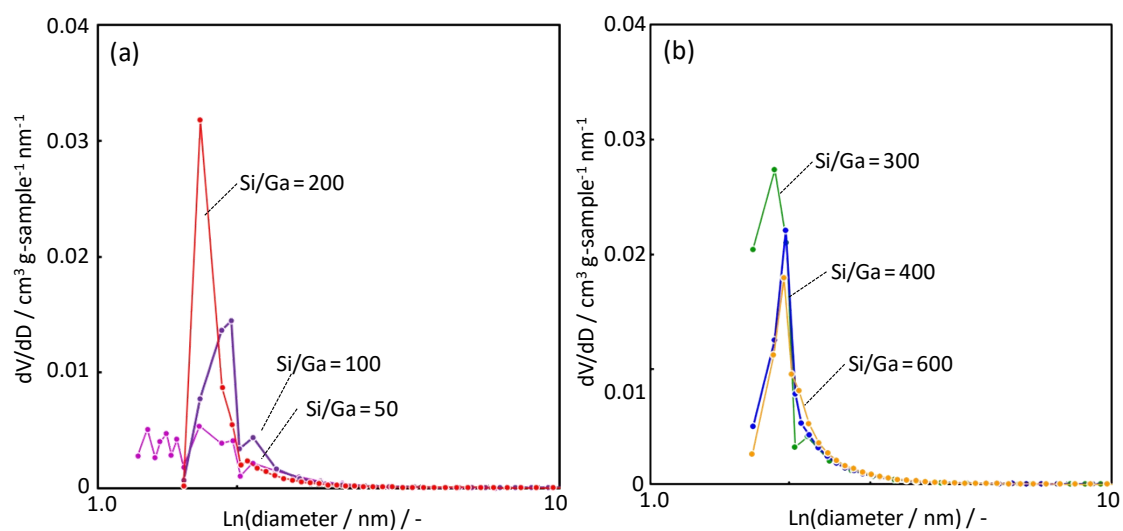

Supplement: RA-013-D3RA01852K-s001 [file RA-013-D3RA01852K-s001.pdf]
